# Supplementary material for: Individual Treatment Trials—Do Experts Know and Use This Option to Improve the Treatability of Mucopolysaccharidosis?
Source: Pharmaceuticals (Basel). 2023 Mar 9;16(3):416. doi: 10.3390/ph16030416 (PMC10058611; doi:10.3390/ph16030416)

All Survey Participants					
**No.**	**City**	**Country**	**Affiliated Department**	**Primary Speciality**	**Patients under treatment**
1	Venice	Italy	Center for Rare Diseases	inherited metabolic diseases	21 - 50
2	Mainz	Germany	Pediatrics, Cardiology, inherited Cardiomyopathies	Cardiology	> 100
3	Heidelberg	Germany	Genetics	Medical Genetics	51 - 100
4	Salzburg	Austria	Pediatrics	inherited metabolic diseases	11 - 20
5	Weilmunester	Germany	Pediatrics	neuropediatrics	> 100
6	Barcelona	Spain	n.a.	n.a.	21 - 50
7	Stanford	USA	Pediatrics	Endocrinology and metabolism	11-20
8	Ermesinde	Portugal	Pediatrics	Pediatrics	< 10
9	Porto	Portugal	Pediatrics	Inherited metabolic diseases	21 - 50
10	Monza	Italy	Pediatrics	Inherited metabolic diseases	51 - 100
11	Stuttgart	Germany	Pediatrics	Suregeon	51 - 100
12	Teo	Spain	Pediatrics	Inherited metabolic diseases	11 - 20
13	Coimbra	Portugal	Pediatrics	n.a.	< 10
14	Berlin	Germany	Endocrinology	Endocrinology, Internal medicine	< 10
15	Innsbruck	Austria	Pediatrics	Inherited metabolic diseases	< 10
16	Vienna	Austria	Pediatrics	inherited metabolic diseases	11 - 20
17	Quito	Ecuador	genetics	clinical genetics	< 10
18	Buenos Aires	Argentina	Pediatrics	IEM	21 - 50
19	Medelin	Colombia	Pediatrics	Neuropediatrics	11 - 20
20	Belem	Brazil	Genetics	Laboratory Assitent	n.a.
21	Rio de Janeiro	Brazil	Genetics	clinical genetics, pediatrics	21 - 50
22	Rio de Janeiro	Brazil	Genetics	Medical Genetics	< 10
23	Lima	Peru	Pediatrics	IEM	11 - 20
24	Vienna	Austria	Internal medicine	inherited metabolic diseases	21 - 50
25	Milan	Italy	Pediatrics	IEM	21 - 50
26	Hannover	Germany	Pediatrics	LSD	21 - 50
27	Bochum	Germany	Pediatrics	Neuropediatrics	< 10
28	Reutlingen	Germany	Pediatrics	Inherited metabolic diseases	11 - 20

Participants Included in the Analysis	
**Country/ region**	**Participants**
**Italy**	**3**
**Germany**	**8**
**Austria**	**4**
**Spain**	**2**
**USA**	**1**
**Portugal**	**3**
**Ecuador**	**1**
**Argentina**	**1**
**Colombia**	**1**
**Brazil**	**2**
**Peru**	**1**
**Europe**	20
**USA**	1
**South America**	6
**total**	**27**

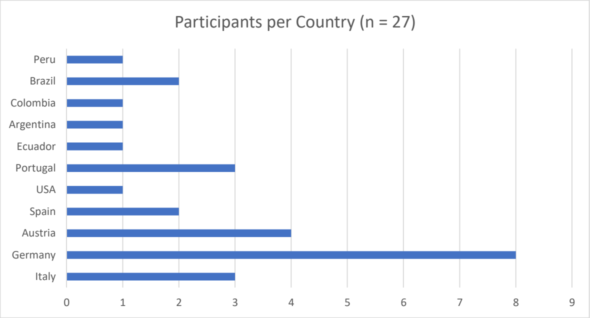

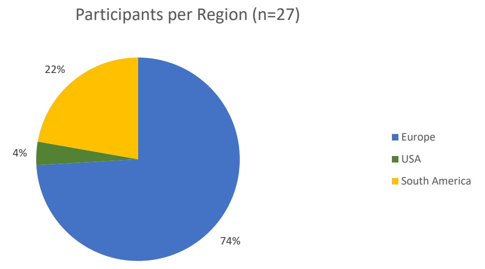

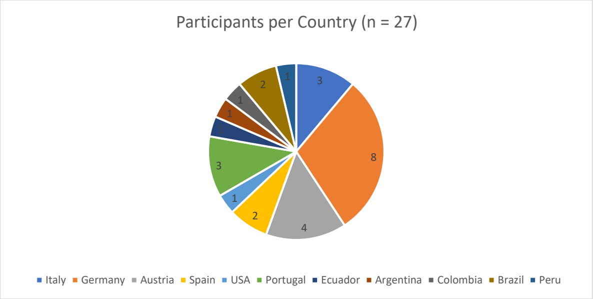

Supplement: Supplementary file 1 [file pharmaceuticals-16-00416-s001.zip › pharmaceuticals-2256547-supplementary.pdf]
